# Enhancing Recovery in Geriatric Spine Surgery: From Risk Prediction to Patient Preparation

**DOI:** 10.1111/ggi.70250

**Published:** 2025-11-05

**Authors:** Chao‐Chun Huang

**Affiliations:** ^1^ Department of Physical Medicine and Rehabilitation Far Eastern Memorial Hospital New Taipei City Taiwan; ^2^ Department of Physical Therapy and Assistive Technology National Yang Ming Chiao Tung University Taipei Taiwan

To the Editor,

1

We commend Mui et al. for addressing an important and underexplored question: whether surgery for lumbar spinal stenosis can truly restore locomotive function in older adults [1]. Their findings—that postoperative GLFS‐25 scores improved from 45.5 to 22 but remained higher than the healthy cohort's 3 points—highlight both the benefit and the limitation of surgery in this population. Their study advances understanding of mobility outcomes and prompts an important discussion on optimizing surgical readiness in older adults. To build upon their findings, we wish to offer three constructive points and propose a forward‐looking agenda for research and practice.

First, the proposed Geriatric Locomotive Function Scale‐25 (GLFS‐25) score of 37 as a clinical cutoff for surgical timing warrants cautious interpretation [1]. Because it was derived from a small sample of only 42 patients, the cutoff may reflect study‐specific variation rather than a generalizable threshold. Reliable clinical cutoffs require large, prospective studies with external validation [2]. Therefore, the GLFS‐25 cutoff of 37 should be viewed not as a clinical directive, but as a valuable hypothesis that now requires robust validation.

Second, the study identifies low Prognostic Nutritional Index (PNI) and female sex as predictors of poor outcomes [1]. While clinically relevant, these factors are also strong surrogates for a larger, unmeasured condition: frailty. Malnutrition is a well‐established contributor to poor surgical recovery, including postoperative delirium [3]. Frailty—a state of reduced physiological reserve and resilience—is a powerful independent predictor of adverse surgical outcomes, including poor functional recovery and the need for post‐discharge facility care [4]. The association between PNI, sex, and outcomes is likely explained by the multidimensional vulnerability captured by frailty. Future prognostic models should incorporate validated, easy‐to‐use tools like the modified 5‐item Frailty Index (mFI‐5) to yield a more comprehensive and clinically actionable assessment of patient risk.

Finally, and most importantly, we advocate for a paradigm shift from simply predicting risk to actively managing it. The finding that surgery alone does not restore patients to the level of their healthy peers underscores the need for preoperative optimization [1]. Rather than using risk factors like a high GLFS‐25 score or low PNI as static predictors of a poor outcome, they should serve as actionable triggers for intervention. This proactive strategy, known as “prehabilitation,” involves a multimodal program of targeted exercise, nutritional supplementation, and psychological support to enhance a patient's physiological resilience before surgery [5]. While evidence in orthopedic and oncologic surgery supports this strategy, its role in spine surgery remains largely unexplored and represents an important opportunity for future research. Investigating the benefits of prehabilitation may ultimately have a greater impact on outcomes than refining surgical timing alone.

In conclusion, the work by Mui et al. is a valuable catalyst for advancing the care of elderly patients with lumbar spinal stenosis. We believe the path forward requires a research agenda focused on three key areas: (1) large‐scale, prospective validation of prognostic tools like the GLFS‐25 cutoff score; (2) the development of comprehensive risk models that integrate frailty assessments; and (3) randomized controlled trials to evaluate the efficacy of targeted prehabilitation programs for high‐risk patients. This approach will help shift the clinical focus from simply timing an intervention to actively creating the best possible candidate for surgery, thereby helping transform spine surgery for frail older adults from reactive to preventive care—an aspiration well aligned with the goals of geriatric rehabilitation.

## Ethics Statement

The author has nothing to report.

## Consent

The author has nothing to report.

## Conflicts of Interest

The author declares no conflicts of interest.

## Data Availability

Data sharing not applicable to this article as no datasets were generated or analysed during the current study.
